# Editorial: Mobile health application in addictive disorders therapy

**DOI:** 10.3389/fpsyt.2024.1360744

**Published:** 2024-02-02

**Authors:** Sasan Adibi, Saeideh Valizadeh-Haghi, Yasser Khazaal, Shahabedin Rahmatizadeh

**Affiliations:** ^1^ School of Information Technology, Deakin University, Geelong, VIC, Australia; ^2^ Department of Medical Library and Information Science, School of Allied Medical Sciences, Shahid Beheshti University of Medical Sciences, Tehran, Iran; ^3^ Department of Psychiatry, Lausanne University Hospital and Lausanne University, Lausanne, Switzerland; ^4^ Department of Health Information Technology and Management, School of Allied Medical Sciences, Shahid Beheshti University of Medical Sciences, Tehran, Iran

**Keywords:** addictive disorders, mHealth, electronic health literacy, digital health, addiction, addiction therapy, non-medical addiction therapy

## Introduction

Addictive disorders cast a formidable shadow on global public health, demanding innovative and effective solutions. In response to this urgent need, the fusion of mobile health (mHealth) technologies with addiction therapy has emerged as a promising avenue for understanding, preventing, and treating addictive behaviors (Mallorquí-Bagué et al.; Hrynyschyn et al.; Charron et al.; Serre et al.; Zhang et al.; Muhlner et al.; Mide et al.). This Research Topic delves into the expansive realm of possibilities that digital interventions and online resources bring to the landscape of addictive disorders therapy. This editorial provides an overview, outlining the historical context, the evolution of technology in healthcare, and the specific opportunities that mobile health introduces to the field of addiction therapy.

## Historical context

The interplay between health and technology is not a recent phenomenon. Throughout history, from the advent of the printing press to the development of telemedicine, technology has continually shaped how we perceive, manage, and treat health-related issues. In the context of addictive disorders, the historical trajectory reflects a shift from traditional face-to-face therapeutic modalities to the integration of digital solutions. Understanding this historical context is pivotal to contextualizing the current wave of mHealth interventions and their potential implications for addictive disorders therapy.

### Evolution of technology in healthcare

The evolution of technology in healthcare has followed a trajectory from analog systems to the digitization of health records, and now, the integration of mobile applications for real-time monitoring and intervention. The advent of smartphones, coupled with advances in connectivity and data analytics, has transformed how individuals access, manage, and engage with healthcare information (1). This evolution is especially relevant in addiction therapy, where personalized, on-the-go interventions can address the dynamic and evolving nature of addictive behaviors.

### Mobile health in addiction therapy

The unique affordances of mobile health bring a paradigm shift to addiction therapy. The ubiquity of smartphones allows for continuous, unobtrusive monitoring of behaviors and triggers. Mobile applications offer an interactive platform for users to engage with therapeutic content, ranging from cognitive-behavioral interventions to immersive virtual reality experiences. As we explore the diverse array of mHealth applications, it becomes evident that this technology has the potential to transcend geographical and temporal barriers, providing a scalable and accessible approach to addiction therapy.

### Challenges and opportunities

While the integration of mobile health in addiction therapy holds immense promise (2), it is not without challenges (3). One of them are related to app usage and engagement. Issues (4) of digital literacy (5), privacy concerns, and the need for personalized interventions that resonate with diverse populations require careful consideration. Additionally, the rapid pace of technological advancements necessitates ongoing evaluation and adaptation of interventions to ensure relevance and efficacy. Recognizing these challenges as opportunities for growth and refinement is crucial in steering the field toward sustainable and impactful solutions.

The historical context and the evolution of technology in healthcare provide a backdrop for understanding the transformative potential of mHealth in addressing the complexities of addiction. As we navigate this intersection, it is imperative to critically examine the challenges and embrace the opportunities that mobile health brings to the forefront of addiction therapy. This Research Topic aims to contribute to this evolving narrative by presenting a collection of studies that unravel the multifaceted dimensions of mHealth interventions in the realm of addictive disorders.

## State-of-the-art technologies

The papers published within this Research Topic represent a rich tapestry of state-of-the-art technologies applied to addictive disorders therapy. At the forefront is the exploration of web-based cognitive-behavioral therapy (CBT4CBT) for cocaine use disorder (Mallorquí-Bagué et al.). This approach exemplifies the fusion of evidence-based therapeutic interventions with the accessibility and scalability afforded by online platforms. Similarly, virtual reality-based tools for alcohol prevention showcase how immersive experiences can influence perceptions and behaviors related to substance use. Additionally, the introduction of app-delivered digital therapeutic programs specifically designed for methamphetamine use disorder demonstrates the adaptability and versatility of technology in addressing diverse addictive behaviors (Hrynyschyn et al.; Muhlner et al.).

## Methodologies

Diverse methodologies characterize the studies included in this Research Topic. Randomized controlled trials (RCTs) assess the effectiveness of digital interventions, offering valuable insights into the comparative efficacy of these innovative approaches. Simultaneously, qualitative studies provide a nuanced understanding of user experiences (Mallorquí-Bagué et al.; Hrynyschyn et al.; Charron et al.) shedding light on the acceptability and feasibility of interventions in real-world settings. This methodological diversity enhances the robustness of our collective understanding, ensuring a comprehensive exploration of the complex landscape of mHealth for the treatment of addictive disorders.

## Trends and future work

An examination of the trends within this Research Topic reveals the evolving landscape of mobile health interventions for addictive disorders. Personalization emerges as a key theme, with interventions tailored to individual needs, preferences, and readiness for change. Ecological momentary assessments, facilitated by mobile technology, enable real-time monitoring of behaviors and triggers, providing a granular understanding of the dynamics of addiction (Zhang et al.). Looking to the future, the integration of artificial intelligence (6) holds promise for optimizing interventions based on continuous learning from user interactions. Virtual reality, with its potential to simulate and modify real-world scenarios, such as training for alternative behaviors in craving triggered contexts (7, 8), presents an exciting frontier for immersive and contextually rich therapeutic experiences. These trends collectively propel the field toward increasingly sophisticated and impactful interventions.

## Conclusion

In conclusion, this Research Topic stands as a testament to the dynamic and evolving landscape of research at the intersection of mobile health and addictive disorders therapy. The varied contributions showcased in this Research Topic underscore the potential of digital technologies in revolutionizing how we approach and treat addiction. However, it is crucial to acknowledge that the journey from research findings to practical implementation in healthcare settings requires collaborative efforts. Bridging this gap will necessitate ongoing dialogue among researchers, clinicians, policymakers, and technology developers to ensure that the benefits of these advancements reach those who need them most (9).

## Author contributions

SA: Writing – original draft, Writing – review & editing. SV: Writing – original draft, Writing – review & editing. YK: Writing – original draft, Writing – review & editing. SR: Writing – original draft, Writing – review & editing.
